# Maternal and Neonatal Outcomes in Pregnant Women With Chronic Hypertension: A Retrospective Study of 50 Cases

**DOI:** 10.7759/cureus.70316

**Published:** 2024-09-27

**Authors:** Bibi Sarah Yousofzai, Khalida Walizada, Rida Mehmood, Rana Beloulou Latoui, Muhammad Subhan, Ernette Espiegle, Freshta Khoshbakht, Lakshmi Venkata Sharmista, Ruqiya Bibi, Muaz Shafique Ur Rehman

**Affiliations:** 1 Obstetrics and Gynecology, Be Team Cure International Hospital, Kabul, AFG; 2 Neurological Surgery, Ali Abad Teaching Hospital, Kabul, AFG; 3 Medicine, Rawalpindi Medical University, Islamabad, PAK; 4 Obstetrics and Gynecology, Centre Hospitalo Universitaire (CHU) Ibn Rochd Hospital, Annaba, DZA; 5 Medicine, Allama Iqbal Medical College, Lahore, PAK; 6 Internal Medicine, Faculty of Medicine and Pharmacy, State University of Haiti, Port-au-Prince, HTI; 7 Medicine, Ghalib Medical University, Herat, AFG; 8 Obstetrics and Gynecology, Kanyakumari Medical Mission Research Center, Hyderabad, IND; 9 Internal Medicine, Jinnah Hospital, Lahore, Lahore, PAK

**Keywords:** aki outcome, a retrospective study, chronic hypertension, chronic hypertension superimposed preclamsia, complication of hypertension in preganancy, gestational hypertension, gestational hypertension pregnancy, hypertension in pregnancy, preterm birth (pb), preterm labour

## Abstract

Background

Chronic hypertension (CHTN) has been associated with significant maternal and neonatal complications. The goal of this research study is to assess outcomes and management strategies among pregnant women living with CHTN.

Methods

From December 2019 to December 2020, 50 pregnant women diagnosed with CHTN before or at 20 weeks gestation at Cure International Hospital, Kabul, Afghanistan, underwent retrospective analysis using data obtained from patient records, such as demographic details, clinical history notes, laboratory test findings, imaging results, management protocols, and maternal and neonatal outcomes.

Results

Of the 50 patients, 17 (34%) developed superimposed preeclampsia. Maternal complications included superimposed preeclampsia in 17 (34%), placental abruption in 14 (28%), gestational diabetes in 15 (30%), and acute kidney injury in one (2%). Neonatal complications included preterm birth (12; 24%), low birth weight (12; 24%), small for gestational age (10; 20%), and perinatal mortality (5; 10%). Management strategies revealed that 38 (75%) patients received labetalol, while 12 (25%) were treated with methyldopa.

Conclusions

CHTN during gestation can result in significant maternal and neonatal complications, and adherence to management guidelines is critical to improve outcomes. More studies are necessary to develop optimal treatment protocols and preventive measures.

## Introduction

Chronic hypertension (CHTN) in pregnancy can be defined as elevated pre-conceptional or early gestational hypertension that is diagnosed before 20 weeks gestation and sometimes persists postpartum, impacting 1-5% of pregnancies, increasing risks such as superimposed preeclampsia, placental abruption, and adverse neonatal outcomes such as preterm birth or fetal growth restriction (FGR) [1]. CHTN is more prevalent among women of advanced maternal age, those who already have preexisting cardiovascular conditions, obesity, or diabetes, or those with a family history of hypertension [2]. Hypertensive disorders in pregnancy affect 5-10% of pregnancies worldwide, and CHTN is one of the significant contributors to maternal morbidity and mortality [3]. Pathophysiologically, preeclampsia causes vasoconstriction, endothelial dysfunction, and impaired placental perfusion - increasing the pregnant woman’s risk for preeclampsia and fetal growth issues [4,5]. Hypertensive disorders account for 10-15% of maternal deaths worldwide, while in the United States, preeclampsia complications arise in 5-8% of pregnancies (with higher rates among women with CHTN), similar to European figures reporting superimposed preeclampsia rates of 10-30% [5,6]. Essential diagnostic tools include blood pressure monitoring, renal function tests, and proteinuria assessments [7-10]. As part of their overall management strategies, doctors focus on blood pressure control, fetal monitoring, and timely deliveries, using preventive strategies like low-dose aspirin for preeclampsia prevention [10-13]. This study seeks to assess maternal and neonatal outcomes among women diagnosed with CHTN, explicitly focusing on the incidence of preeclampsia superimposed with CHTN, maternal complications, and neonatal health outcomes, as well as gaps in management practices to recommend improvements [13-17].

## Materials and methods

Study design

This retrospective study, conducted at Cure International Hospital, Kabul, Afghanistan, from December 2019 to December 2020, examined women with CHTN and their outcomes. The study was approved by the Institutional Review Board of the Ministry of Public Health of Afghanistan (IRB code No. 2023/IRB/001), data were de-identified, and individual consent was waived. It followed the ethical standards of the Declaration of Helsinki.

Study participants

A total of 50 pregnant women diagnosed with CHTN either before conception or during the first 20 weeks of gestation were included in the study. The inclusion and exclusion criteria used in this study are presented in Table 1.

**Table 1:** Inclusion and exclusion criteria for study participants CHTN, chronic hypertension

Criteria	Details
Inclusion criteria	Pregnant women diagnosed with CHTN before or at 20 weeks of gestation
	Women who were receiving prenatal care and hypertensive management
Exclusion criteria	Patients diagnosed with hypertension after 20 weeks of gestation (gestational hypertension)
	Patients with incomplete or missing medical records

Data collection

Data were collected from patient records, including demographic details, clinical history, laboratory findings, imaging results (primarily ultrasound), management protocols, and maternal and neonatal outcomes. Specific outcomes included the incidence of superimposed preeclampsia, maternal complications (such as placental abruption, preterm labor, etc.), and neonatal complications (such as low birth weight, preterm birth, etc.). Maternal data included age, BMI, blood pressure, prenatal visits, treatment protocols (antihypertensive medications), complications such as superimposed preeclampsia, placental abruption, and delivery outcomes. Neonatal data included birth weight, gestational age, Apgar score, neonatal intensive care unit (NICU) admissions, perinatal mortality, and congenital anomalies. Table 2 summarizes the history, physical examination, and initial assessments of all 50 patients, including lab investigations and ultrasound.

**Table 2:** History and physical examination of involved patients

Parameters	Total patients studied	Number of patients receiving management	Percentage (%)
History	50	50	100%
Physical examination	50	50	100%
Lab investigation	50	40	80%
Ultrasound	50	48	96%
Management	50	50	100%

Definitions

Operational definitions of different terminologies used in our current research are described in Table 3.

**Table 3:** Operational definitions

Term	Definition	References
Chronic hypertension	Hypertension diagnosed before pregnancy or before 20 weeks of gestation	[1,2]
Superimposed preeclampsia	Preeclampsia that develops in a woman with preexisting chronic hypertension, defined by new-onset proteinuria or end-organ dysfunction	[2,3]
Severe hypertension	Blood pressure greater than 160/110 mmHg	[2,3]
Gestational age	Time in weeks from the first day of the last menstrual period	[5]
Birth weight	Weight of the neonate immediately after birth	[6]
Length-for-gestational age	Classification based on fetal length compared to standardized growth curves	[7]
Preterm	Birth before 37 weeks of gestation	[3,4]
Low birth weight	Birth weight less than 2,500 grams	[3,4]
Appropriate for gestational age	Neonates with birth weight between the 10th and 90th percentiles for their gestational age	[3,4]
Small for gestational age	Neonates with birth weight below the 10th percentile for their gestational age	[4,5]
Large for gestational age	Neonates with birth weight above the 90th percentile for their gestational age	[4,5]
Perinatal mortality	Death of a fetus after 22 weeks of gestation or a neonate within the first seven days of life	[5]

Statistical analysis

Descriptive statistics were utilized to summarize the data. Categorical variables such as maternal age and blood pressure were reported using proportions and percentages.

## Results

**Figure 1:**
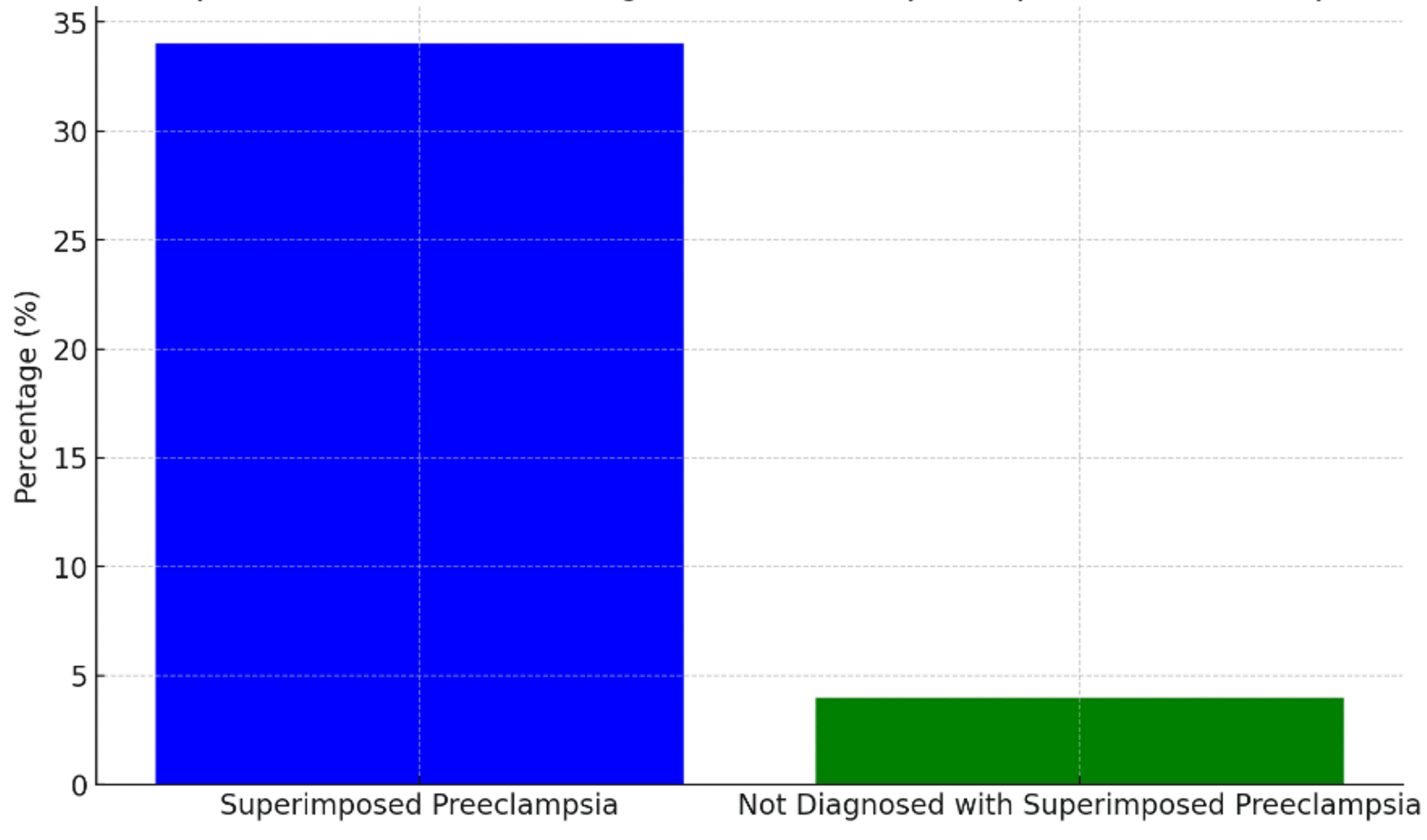
Comparison of patients diagnosed with superimposed preeclampsia

In our study, we extensively discussed several critical parameters related to the management and outcomes of CHTN in pregnancy. Specifically, we examined maternal complications, neonatal complications, and prenatal care practices, including the timing of delivery for women with hypertension. Our study also focused on the management strategies for CHTN with superimposed preeclampsia, highlighting the importance of appropriate treatment and monitoring. Additionally, we addressed the prevention of recurrence in preeclampsia, exploring approaches to mitigate the risks and improve outcomes for affected individuals. Superimposed preeclampsia affects 13-40% of pregnant women with CHTN and is linked with higher rates of adverse outcomes. Our study involved 50 patients, and 17 were diagnosed with superimposed preeclampsia (34% of our cohort), in line with its highest end reported elsewhere. Notably, two patients out of our study did not fall under this diagnosis - which equated to 4% (two patients) - representing a significantly lower undiagnosed rate than traditional reporting methods. Figure 1 depicts the comparison of the percentage of patients diagnosed with superimposed preeclampsia versus those not diagnosed.

Maternal complications

**Figure 2:**
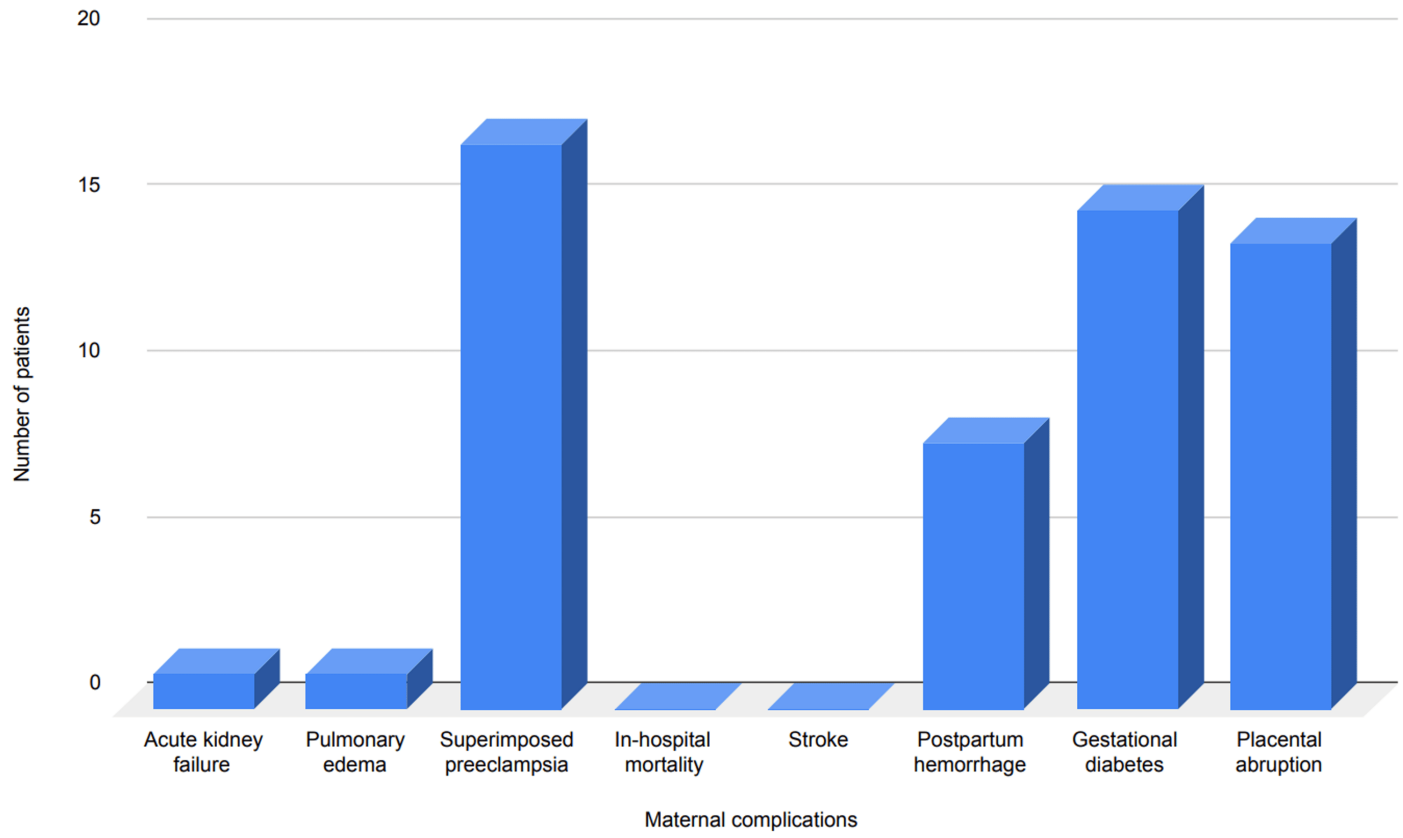
Maternal complications in the included patients

From the data on 50 patients, both the frequency (n) and percentage (%) of maternal complications were assessed. Acute kidney failure occurred in one patient (2%), while pulmonary edema was also observed in one patient (2%). Superimposed preeclampsia was diagnosed in 17 patients (34%), and placental abruption occurred in 14 patients (28%). Gestational diabetes was identified in 15 patients (30%). No cases of in-hospital mortality or stroke/cerebrovascular complications were reported. Additionally, postpartum hemorrhage was observed in eight patients (16%). Figure 2 shows the overall data on different maternal complications.

Neonatal complications

In our retrospective approach of 50 pregnant cases, numerous fetal and neonatal complications were noted. Of the total cases, 12 patients (24%) were classified as preterm, delivering before 37 weeks of gestation, while the majority, 38 patients (76%), were term deliveries, indicating they reached or exceeded the standard gestational age. Regarding birth weight, 12 neonates (24%) were categorized as having low birth weight, weighing less than 2,500 grams, whereas 38 neonates (76%) were classified as having normal birth weight, weighing 2,500 grams or more. These findings highlight a significant proportion of term deliveries and normal birth weights, reflecting overall favorable gestational age and neonatal weight outcomes within the study population. Perinatal mortality occurred in five cases (10%) - two to four times more likely than that seen in the general population. Preterm birth before 37 weeks gestation with birth weight less than 2,500 grams occurred in 12 (24%), often necessitating admission into NICU units for care. Small for gestational age was present in 10 neonates (20%), reflecting restricted fetal growth; congenital malformations were present in one case (2%), such as congenital cardiac malformations, esophageal atresia, and hypospadias. Significant outcomes of neonatal parameters for our study are described well in Table 4 and Table 5.

**Table 4:** Size for gestational age classification

Size for gestational age	n (%)
Small for gestational age	10 (20%)
Appropriate for gestational age	39 (78%)
Large for gestational age	1 (2%)

**Table 5:** Neonatal and fetal complications NICU, neonatal intensive care unit

Complication	n (%)
Perinatal mortality	5 (10%)
Congenital malformations	1 (2%)
Admission to the NICU	12 (24%)

Prenatal care and management

Preconception and prenatal care are integral parts of maternal health, particularly for women living with chronic illnesses like hypertension [1,2]. Preconception care should include consultation with maternal-fetal medicine specialists who can offer counseling and management both before conception and during gestation [3,4]. Counseling on risk factors, assessing secondary causes of hypertension, and conducting laboratory investigations such as creatinine levels or urine protein/creatinine ratios may all play a part [3,4]. Additional measures could include cardiac evaluations, weight loss in overweight or obese patients, quitting smoking, and managing blood pressure efficiently [3,4]. Prenatal care requires routine clinical blood pressure assessment and general physical exams, including cardiopulmonary auscultation [4,5]. Routine prenatal investigations such as measuring creatinine levels, urinary protein content, and thyroid function testing are crucial parts of care [5,6]. Accurate gestational dating through first-trimester ultrasound and an in-depth fetal anatomical survey between 18 and 20 weeks is critical, given the increased risks for growth restriction and preterm delivery associated with CHTN patients [5,6]. Preeclampsia prevention should also include taking low-dose aspirin after 12 weeks of gestation to lower its risks [4-6]. Our study further evaluated these prenatal care parameters by reviewing data: 100% of patients (n = 50) underwent a comprehensive history and physical exam; 84% (42 cases) received routine prenatal laboratory testing; 96% (48 cases) underwent ultrasound examination; none had their BMI measured; and 72% (36 cases) received a low-dose aspirin prescription. These results reflect compliance with guidelines for preconception and prenatal care.

Management of severe hypertension

Severe maternal hypertension, defined as having a systolic blood pressure exceeding 160 mmHg or diastolic blood pressure exceeding 110 mmHg, must be addressed promptly to avoid severe maternal complications such as cerebrovascular, cardiac, and renal events [5,6]. When it comes to non-severe hypertension, management varies based on individual patient factors: in cases without antihypertensive therapy and no end-organ damage, the target blood pressure range should remain 120-150/80-95 mmHg, while in those on antihypertensive treatment, patients without end-organ damage treatment should remain 130/85 mmHg, respectively. When treating patients with end-organ damage, antihypertensive therapy may become necessary; antihypertensive treatment is advised if either exceeds 150 mm or diastolic exceeds 100 mmHg [1-6]. Our study of 50 patients revealed that three patients (6%) with severe hypertension did not adhere to recommended blood pressure targets. However, 47 patients (94%) were managed correctly, highlighting the overall success in maintaining appropriate blood pressure control in most cases.

**Figure 3:**
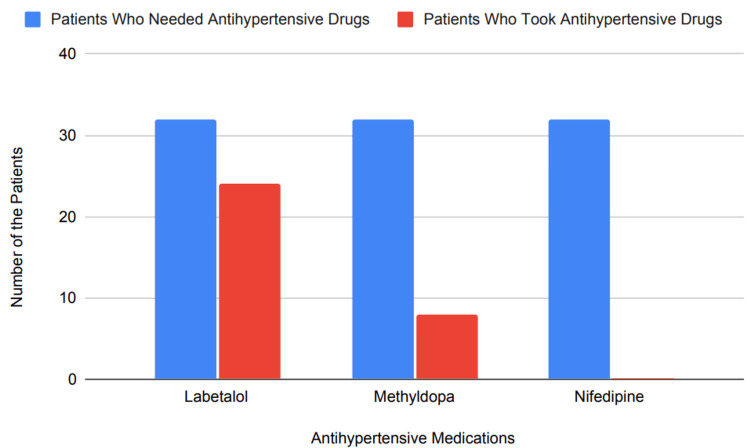
Medication utilization for CHTN management in pregnancy CHTN, chronic hypertension

Management of non-severe hypertension during gestation requires following specific guidelines to ensure maternal and fetal safety [5,6]. According to recent recommendations, medications including labetalol (up to 2,400 mg daily, divided and taken twice per day, with possible contraindications for conditions like asthma or heart block), extended-release nifedipine (120 mg per day and not recommended in cases of tachycardia), and methyldopa (3,000 mg daily and taken two or three times a day) may be taken in various combinations as appropriate for each person's particular circumstances [4-6]. Suppose a target blood pressure range of 120-150/80-95 mmHg cannot be achieved with a maximum dose of one antihypertensive. In that case, additional antihypertensives may be added based on overall health considerations and any contraindications [4-6]. Our study of 32 patients who required medication therapy revealed that 24 (75%) received labetalol therapy, eight (25%) received methyldopa, and none (0%) received extended-release nifedipine. Figure 3 shows the graphical distribution of the data mentioned above.

Monitoring fetal well-being in pregnancy

**Figure 4:**
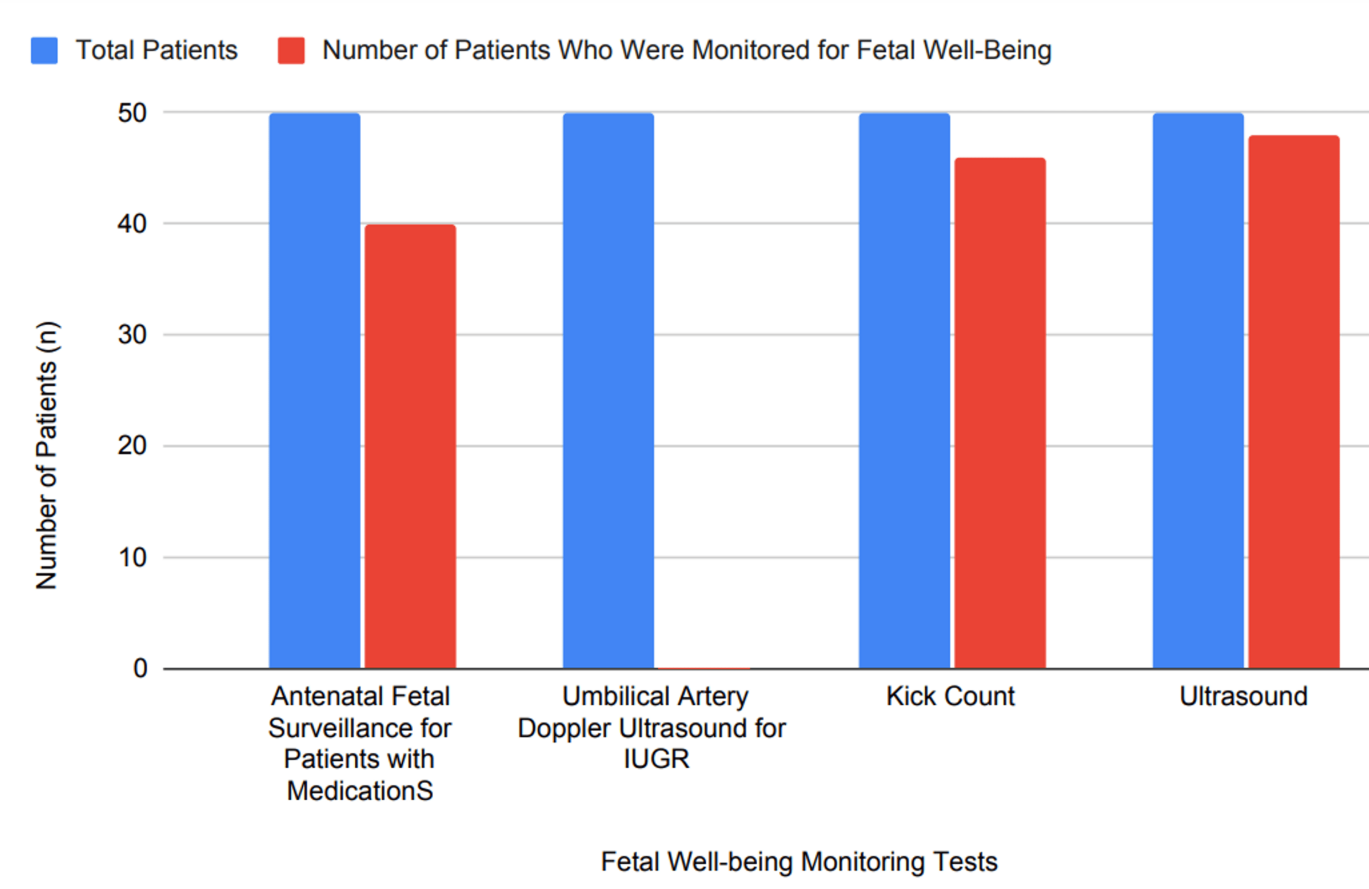
Monitoring of fetal well-being in pregnant patients with CHTN CHTN, chronic hypertension; IUGR, intrauterine growth restriction

According to standard guidelines, the following tests are recommended to assess fetal well-being: maternal evaluation of fetal kick counts; twice-weekly surveillance starting at 32 weeks gestation for patients with well-controlled hypertension, with individualized adjustments in frequency for poorly managed cases. Nonstress tests, biophysical profiles (BPP), or modified BPPs are used, although these do not significantly affect perinatal mortality rates. In pregnancies complicated by FGR, umbilical artery Doppler ultrasound is advised to reduce mortality and mitigate risks associated with FGR [6,7]. Our retrospective analysis of 50 pregnant cases demonstrated adherence to these monitoring practices: 80% (40 cases) were monitored with antenatal fetal surveillance for medication, none (0%) underwent umbilical artery Doppler ultrasound for intrauterine growth restrictions (IUGRs), 92% (46 cases) observed kick counts, and 96% (48 cases) were monitored via ultrasound. Figure 4 depicts the percentage distribution of monitoring of fetal well-being in pregnant patients with CHTN.

Timing of delivery for women with hypertension

**Figure 5:**
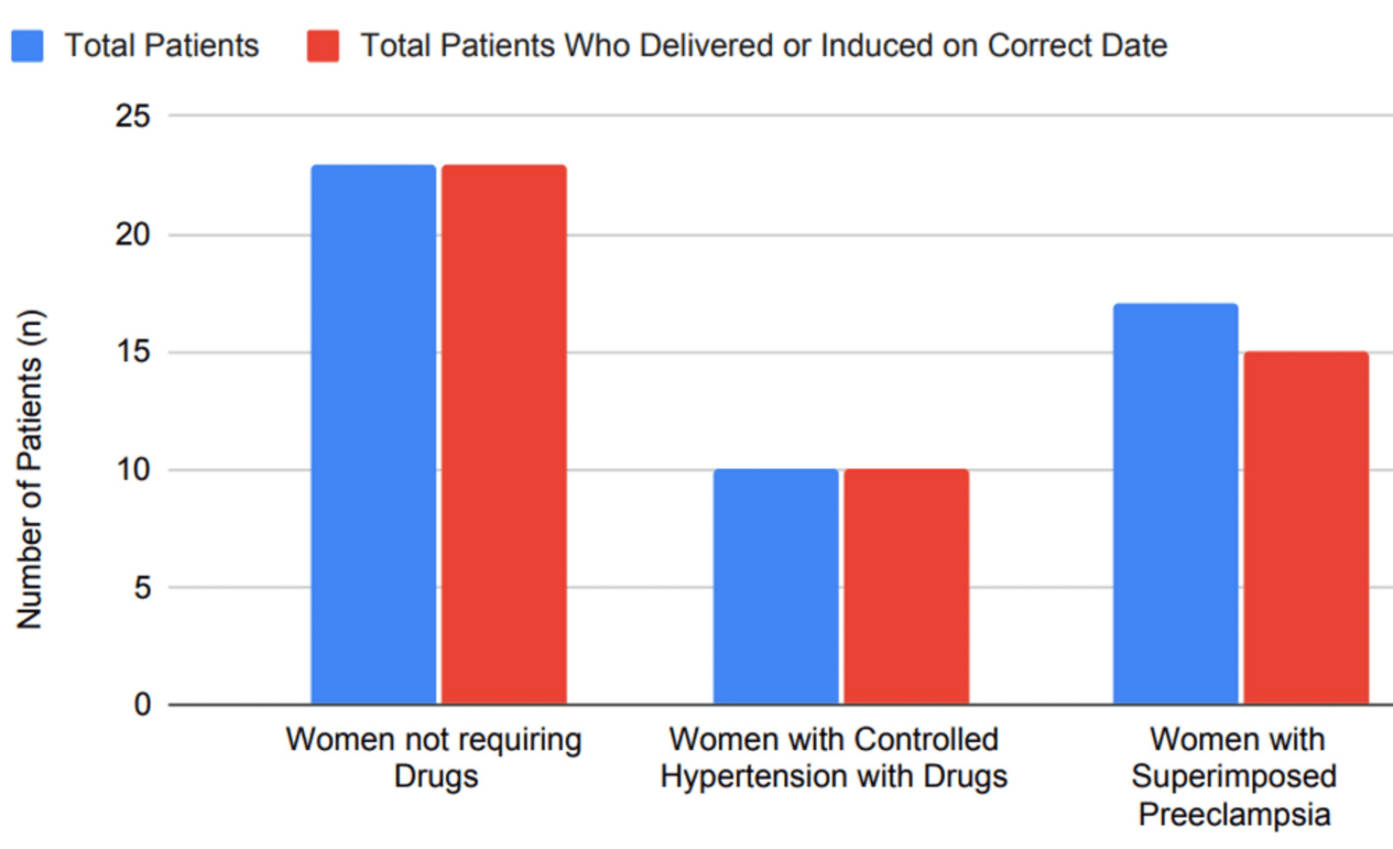
Percentage of patients delivered or induced on the correct date

Delivery timing depends on hypertension management: For women who have effectively managed CHTN, labor should occur between 39 weeks gestation and 39 + 6 weeks [7,8]. The American College of Obstetricians and Gynecologists (ACOG) guidelines specify a delivery timeline for women with high blood pressure that cannot be controlled with medication: >38 to 39 + 6 weeks in gestation for those whose hypertension can be managed through pharmaceutical means and >37 + 0 to 39 + 0 weeks for those needing hypertension treatment with drugs [7,8]. Women experiencing preeclampsia or other pregnancy complications must adjust their delivery date depending on the nature and severity of these complications [8]. Our research indicated that 100% of the 23 women (out of 50 cases) did not require medication for hypertension management. Additionally, 10 women (20%) whose hypertension was effectively controlled with medication either delivered or had their delivery scheduled precisely on or around their due date, or were successfully induced. Among the remaining 17 women (34%) diagnosed with superimposed preeclampsia, timely delivery or induction was necessary, and 15 cases (83%) were successfully delivered as planned, as illustrated in Figure 5.

Management of CHTN with superimposed preeclampsia

**Figure 6:**
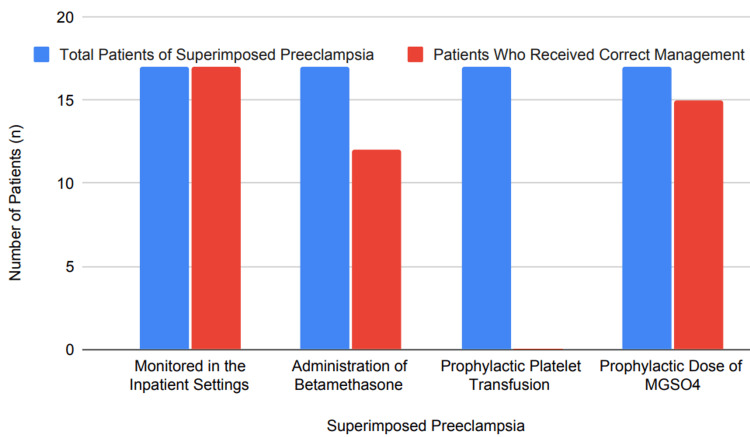
Percentage of correct management for superimposed preeclampsia MGSO4, magnesium sulfate

Superimposed preeclampsia refers to preeclampsia that develops in pregnant women who already had CHTN before gestation [1,3]. Superimposed preeclampsia with severe features is recognized by symptoms including consistently elevated blood pressure despite increasing antihypertensive therapy, thrombocytopenia (platelet count of less than 100,000/microliter), elevated transaminases or severe persistent epigastric or right upper quadrant pain unresponsive to medication and unattributed to another diagnosis, new or worsening renal insufficiency, pulmonary edema, or ongoing cerebral/visual disturbances [2,3]. Current guidelines for treating severe hypertension during gestation include inpatient monitoring to address serious hypertension and its related complications, betamethasone to promote lung maturation if delivery occurs before 34 weeks gestation, and prophylactic magnesium sulfate (MgSO4) use to avoid seizures in severe cases [3,4]. Prophylactic platelet transfusion should only be considered when severe bleeding or significant thrombocytopenia exists [3,4]. Antihypertensive therapy should be intensified immediately, and symptoms like severe pain, renal insufficiency, pulmonary edema, and cerebral/visual disturbances need to be managed quickly [3,4]. Our study of 17 women who developed superimposed preeclampsia showed all were monitored inpatient (100%). Contrariwise, only 12 (70.58%) patients received betamethasone administration; none received prophylactic platelet transfusion (0%), and 15 (88%) were provided preventive MgSO4 doses. Our findings, as depicted in Figure 6, reveal potential areas for improvement regarding current management protocols relating to betamethasone administration and prophylactic platelet transfusion.

Prevention of recurrence in preeclampsia

Preventing preeclampsia from occurring again requires several strategies [5,6]. Low-dose aspirin therapy during gestation may reduce risk in high-risk women [10,12]. If aspirin fails or placental examination reveals extensive decidual inflammation, vasculopathy, or thrombosis occurs despite aspirin treatment [10,12], other medications or therapies, such as weight loss in overweight or obese women, may help lower recurrence risk [5,6]. Techniques that avoid multiple gestations can further lower recurrence risk [5,6].

**Figure 7:**
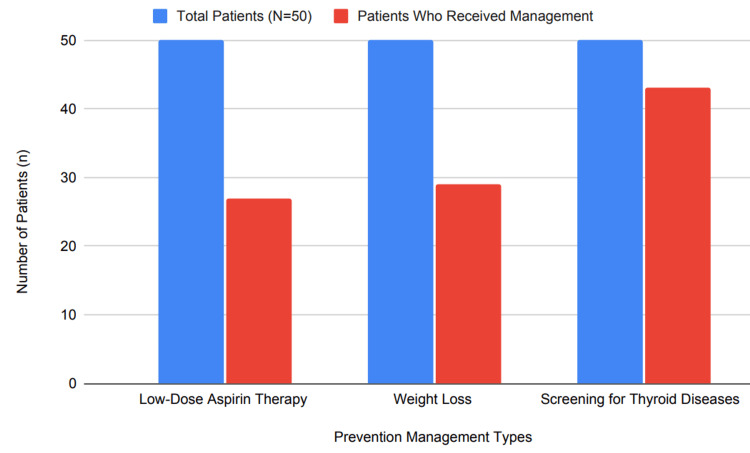
Percentage of patients receiving prevention management

Also, nulliparous women who developed preeclampsia during gestation were twice as likely to develop subclinical hypothyroidism during gestation, with those experiencing recurrence preeclampsia having the highest risk. All patients presenting with symptoms of hypothyroidism should be evaluated; in our study of 50 patients, 27 (54% received low-dose aspirin therapy; none received heparin (0%); 29 (58%) achieved weight loss, while 43 (86% were screened for thyroid diseases), as shown in Figure 7. These data underscored both adherence to preventive measures and areas for improvement regarding managing recurrence prevention measures and areas that need improvement when managing recurrence prevention measures and recurrence prevention measures taken before preeclampsia occurs again during recurrence prevention efforts.

## Discussion

CHTN in pregnancy poses a substantial risk to maternal and neonatal outcomes [1-4]. Our study’s findings concur with existing literature, underscoring an increased risk of complications among this population while providing opportunities to improve management practices. Our study demonstrated an incidence of superimposed preeclampsia at 34%, which aligns with European data, with rates ranging between 13-40% [5-10]. CHTN can quickly worsen, increasing maternal and fetal risks, including eclampsia, hemolysis, elevated liver enzymes, and low platelets (HELLP) syndrome, and increasing maternal mortality rates [3,4]. Our rate of superimposed preeclampsia aligned closely with that reported in the literature, reflecting its complexity during pregnancy.

Furthermore, we found a 28% rate of placental abruption, significantly higher than 10-15% reported elsewhere [5-8]. Placental abruption is an obstetric emergency with serious outcomes, including fetal distress and IUGR. A higher incidence may signal deficiencies in preventative strategies or monitoring practices [5,6]. Gestational diabetes was another significant finding, affecting 30% of our patients and reflecting an increased risk in literature [6,7]. It complicates the management of CHTN by increasing macrosomia risk, cesarean delivery rates, and long-term metabolic issues both for the mother and fetus [6,7].

Concerning neonatal and fetal outcomes, our study reported preterm birth and low birth weight in 24% of cases; these numbers correspond to Bramham et al.’s findings that women with CHTN have an increased risk for these outcomes threefold [10]. Preterm birth and low birth weight have been associated with higher risks of neonatal morbidity, including respiratory distress syndrome and long-term neurodevelopmental delays [6,7]. The perinatal mortality rate among our cohort was 10%, indicative of the grave risks associated with maternal hypertension as well as its need for careful surveillance and intervention. Regarding management strategies, our research revealed that 75% of patients were given labetalol treatment, 25% received methyldopa, and none received extended-release nifedipine. Labetalol’s efficacy and safety profile for managing hypertension during gestation is consistent with current guidelines; however, its use indicates an apparent gap in comprehensive treatment protocols [9]. Extended-release nifedipine can be an effective medication, particularly when other medications do not adequately control blood pressure [9]. A key area for improvement identified in our study was adherence to preventive measures. Despite guidelines recommending low-dose aspirin for preeclampsia prevention, only 72% of patients received it. This gap in compliance may have contributed to the higher rates of superimposed preeclampsia and preterm births observed.

However, none of the patients underwent umbilical artery Doppler surveillance for FGR, which would have helped improve neonatal outcomes by providing earlier intervention opportunities. Our findings aligned with a National Heart, Lung, and Blood Institute (NHLBI) study, demonstrating that early and effective blood pressure control can decrease severe pregnancy complications [13]. Our cohort showed that patients whose blood pressure was well managed experienced fewer complications; however, managing severe hypertension remains challenging, as evidenced by the 6% of patients who did not receive appropriate treatments for severe hypertension. Low utilization of low-dose aspirin in our study exemplifies the need for improved compliance with preventive measures. Studies have demonstrated its ability to substantially lower preeclampsia risks among high-risk populations, yet its usage was less than recommended among our cohort. This gap in preventive care illustrates the need for improved education of healthcare providers on the value of aspirin therapy and fetal monitoring, among other practices.

Furthermore, this research underscores the significance of early diagnosis, aggressive blood pressure control measures, and adherence to preventive measures against future risks. Improved education of healthcare providers on the importance of low-dose aspirin use and comprehensive fetal monitoring could significantly lower rates of adverse maternal and neonatal outcomes. Addressing gaps in current management practices and adhering to clinical guidelines are critical components in improving outcomes for pregnant individuals with CHTN. CHTN can pose substantial threats to both maternal and neonatal health. Our findings emphasize the need for strict blood pressure control, compliance with preventive measures, and continuous fetal monitoring to mitigate risks. Future research should look at barriers to optimal care and strategies that increase guideline adherence to enhance outcomes for this high-risk population.

Limitations

This study has several limitations that should be considered, starting with its relatively small sample size of 50 patients, which may limit its generalizability. At the same time, its retrospective design introduces potential bias and the risk of missing data, which could compromise the accuracy of results. Furthermore, it is only focused on one institution and thus may differ from practices or outcomes at other facilities, potentially restricting results’ applicability to wider settings.

## Conclusions

CHTN during pregnancy presents serious risks to both maternal and neonatal health. Our study highlighted the prevalence of complications such as preeclampsia, placental abruption, and gestational diabetes among pregnant women with CHTN. Neonatal complications were also significantly higher among this population, such as preterm birth, low birth weight, and perinatal mortality rates. These findings demonstrate the importance of following established management guidelines to mitigate risks in this care area. Although effective antihypertensive medication was available, gaps were seen in adhering to treatment protocols, particularly regarding betamethasone administration and prophylactic platelet transfusion for preeclampsia. These gaps provide areas for improvement in clinical practice. Future research should aim to optimize treatment protocols, strengthen preventive measures, and consistently apply guidelines to optimize outcomes for mothers and infants.
